# LC-MS/MS Detection of Karlotoxins Reveals New Variants in Strains of the Marine Dinoflagellate *Karlodinium veneficum* from the Ebro Delta (NW Mediterranean)

**DOI:** 10.3390/md15120391

**Published:** 2017-12-18

**Authors:** Bernd Krock, Julia A. Busch, Urban Tillmann, Francisco García-Camacho, Asterio Sánchez-Mirón, Juan J. Gallardo-Rodríguez, Lorenzo López-Rosales, Karl B. Andree, Margarita Fernández-Tejedor, Matthias Witt, Allan D. Cembella, Allen R. Place

**Affiliations:** 1 Alfred Wegener Institut-Helmholtz Zentrum für Polar- und Meeresforschung, Chemische Ökologie, Am Handelshafen 12, 27570 Bremerhaven, Germany; busch@waddensea-secretariat.org (J.A.B.); urban.tillmann@awi.de (U.T.); allan.cembella@awi.de (A.D.C.); 2 Common Wadden Sea Secretariat, Virchowstr. 1, 26382 Wilhelmshaven, Germany; 3 Chemical Engineering Area, University of Almería, 04120 La Cañada, Spain; fgarcia@ual.es (F.G.-C.), asmiron@ual.es (A.S.-M.); llr288@ual.es (L.L.-R.); 4 Department of Chemical Engineering, Faculty of Engineering, University of Concepción, Concepción 4030000, Chile; juangallardo@udec.cl; 5 Institut de Recerca i Tecnologia Agroalimentàries (IRTA), Ctra Poble Nou km 5.5, 43540 Sant Carles de la Rapita, Tarragona, Spain; karl.andree@irta.es (K.B.A.); margarita.fernandez@irta.cat (M.F.-T.); 6 Bruker Daltonik GmbH, Fahrenheitstr. 4, 28359 Bremen, Germany; Matthias.Witt@bruker.com; 7 Institute of Marine and Environmental Technology, UMCES, Baltimore, MD 21613, USA; place@umces.edu

**Keywords:** phycotoxin, ichthyotoxin, HAB, harmful algal bloom, plankton, Alfacs Bay, Fangar Bay, Mediterranean Sea

## Abstract

A liquid chromatography-tandem mass spectrometry (LC-MS/MS) method was developed for the detection and quantitation of karlotoxins in the selected reaction monitoring (SRM) mode. This novel method was based upon the analysis of purified karlotoxins (KcTx-1, KmTx-2, 44-oxo-KmTx-2, KmTx-5), one amphidinol (AM-18), and unpurified extracts of bulk cultures of the marine dinoflagellate *Karlodinium veneficum* strain CCMP2936 from Delaware (Eastern USA), which produces KmTx-1 and KmTx-3. The limit of detection of the SRM method for KmTx-2 was determined as 2.5 ng on-column. Collision induced dissociation (CID) spectra of all putative karlotoxins were recorded to present fragmentation patterns of each compound for their unambiguous identification. Bulk cultures of *K. veneficum* strain K10 isolated from an embayment of the Ebro Delta, NW Mediterranean, yielded five previously unreported putative karlotoxins with molecular masses 1280, 1298, 1332, 1356, and 1400 Da, and similar fragments to KmTx-5. Analysis of several isolates of *K. veneficum* from the Ebro Delta revealed small-scale diversity in the karlotoxin spectrum in that one isolate from Fangar Bay produced KmTx-5, whereas the five putative novel karlotoxins were found among several isolates from nearby, but hydrographically distinct Alfacs Bay. Application of this LC-MS/MS method represents an incremental advance in the determination of putative karlotoxins, particularly in the absence of a complete spectrum of purified analytical standards of known specific potency.

## 1. Introduction

Members of the marine dinoflagellate genus *Karlodinium* (formerly assigned to *Gymnodinium* F. Stein) have long been implicated as the cause of fish kills and other marine faunal mortalities in wild populations and coastal aquaculture systems around the world [1,2]. Taxonomic identification among this naked (unarmored) dinoflagellate group has been complicated by their lack of thecal plates for tabulation, relatively small size, and plastic morphology. Nevertheless, application of molecular diagnostics has served to resolve many taxonomic inconsistencies, and helped to establish stable species—now eight species defined within this genus. Among these, *K. veneficum* (synonym *Gymnodinium veneficum)* is perhaps the most prominent and notorious species responsible for formation of mass harmful blooms and associated fish kills [2,3,4]. This species tends to remain in the background in low cell abundance (<10^3^ mL^−1^), but occasionally becomes dominant, forming dense blooms with devastating consequences for the health of marine fauna [5,6,7].

**Figure 1:**
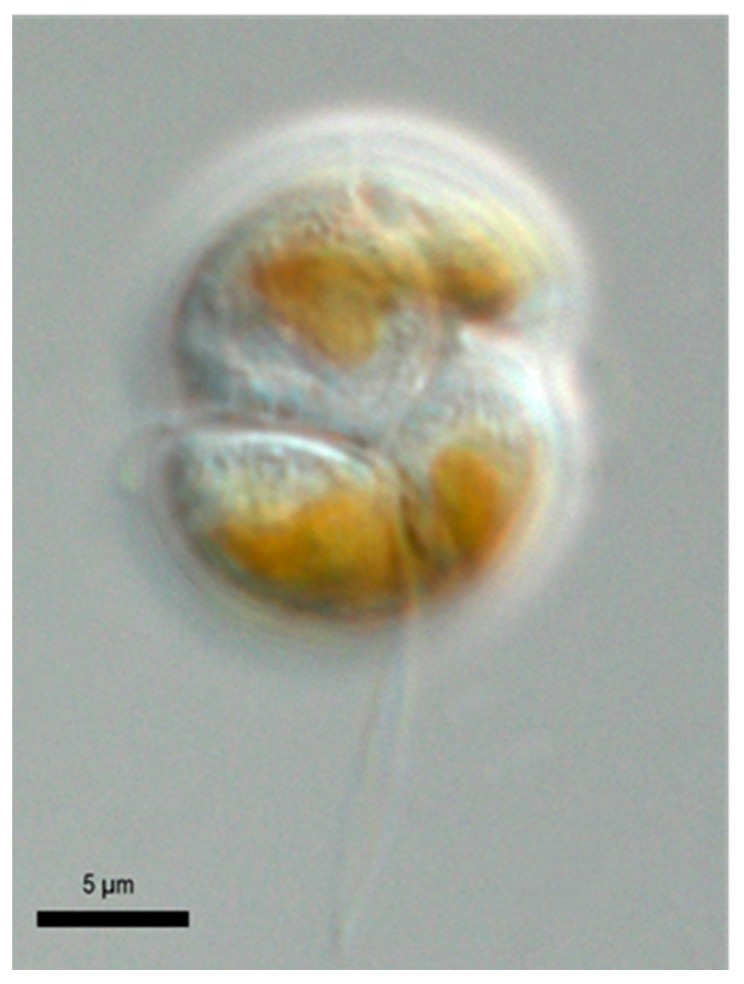
Light microscopic image of *Karlodinium veneficum* strain E11 from Fangar Bay (Ebro Delta).

Several species of *Karlodinium* are reputed to produce potent ichthyotoxins associated with fish and other faunal mortalities [2,5,6,8] but these taxonomic assignments are complicated by previous inconsistencies in identification (see [2] for taxonomic synonyms) and high variability in toxigenicity among strains within a species [9]. In any case, *K. veneficum* is known to produce a unique suite of amphidinol-like polyketide toxins called karlotoxins (KmTxs) (Figure 1) [2]. Karlotoxins have been reported to display a variety of deleterious effects on biological systems, including cellular lysis, damage to fish gills, and immobilization of prey organisms [10]. The cytolytic activity of karlotoxins is modulated by membrane sterol composition, which has been proposed as a mechanism for *K. veneficum* to avoid autotoxicity [2]. In cell and tissue bioassays, karlotoxins exhibit potent hemolytic, cytotoxic, and ichthyotoxic properties [4], whereas in nature, they appear to function as allelochemicals in chemical defense against grazing and/or in prey acquisition [10,11,12].

**Figure 2:**
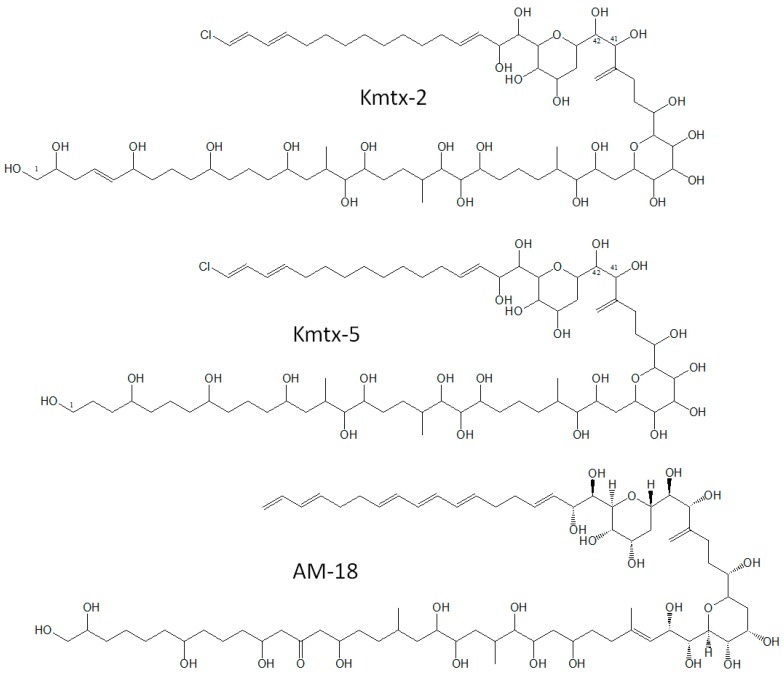
Structures of karlotoxins KmTx-2, KmTx-5, and amphidinol-18 (AM-18).

The current suite of fully characterized karlotoxins comprises seven analogs, with more than half a dozen others assigned a tentative or provisional structure [2,13,14,15]. All analogs have a hairpin-like structure with three distinct regions: a polyol arm that exhibits variable hydroxylation and methylation; a hinge region containing two ether rings; and a lipophilic arm (Figure 2). The lipophilic arm often includes conjugated trienes in amphidinols, but instead a terminal diene in karlotoxins, which can be chlorinated, and gives these compounds their distinctive UV spectra. Among these karlotoxins, two analogs, KmTx-1 [5] and KmTx-2 [16], have been isolated and characterized in sufficient quantities for evaluation of specific potency, e.g., in cell lysis assays, but this is not the case for most of the other analogs. Unfortunately, the lack of a sensitive standardized analytical method for identification and quantitation of karlotoxins has hampered the exploration of specific potency of various analogs, research on allelochemical interactions, the development of alternative bioassay methods, and evaluation of the implications of karlotoxins in seafood safety and regulation.

Standardized protocols are now in place for a number of phycotoxins, such as domoic acid, and polyether toxins, such as spirolides, dinophysistoxins, pectenotoxins, and yessotoxins, based upon liquid chromatography-tandem mass spectrometry (LC-MS/MS). In principle, a robust LC-MS/MS method for karlotoxins should be possible if there is a unique common fragment that defines the group and is detectible at reasonable natural toxin concentrations in dinoflagellate cultures, field phytoplankton, and contaminated seafood or other affected species. As yet, there is no standard chemical analysis for karlotoxins, in spite of an initial attempt [17]. The absence of commercially available analytical standard for any karlotoxins is certainly a major constraint on method development. In spite of this, significant progress can be made with purified (but uncertified) karlotoxins (e.g., KcTx-1, KmTx-2, 44-oxo-KmTx-2, KmTx-5) and unpurified extracts of bulk cultures of *K. veneficum* strains. The overarching objective of this work is to further the development of reliable standardized detection and identification methods for karlotoxins for food safety and research.

## 2. Results

### 2.1. Species Identification

**Figure 3:**
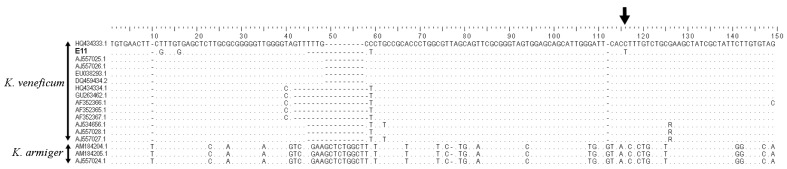
Alignment showing a large indel within the range of bases 40 to 60, where three distinct geno-groups are evident. A conserved C/T transition among strain E11 from Fangar Bay which was used in this study is indicated by the arrow. Dots indicate conserved bases in reference to the *K. veneficum* sequence shown at the top, and dashes indicate gaps inserted for alignment.

Genetic analysis and sequence alignment clearly identified strain E11 as *K. veneficum* (Figure 3). Among *K. veneficum* strains of this study there are three main geno-groups that can be distinguished by a large insertion/deletion (indel) region (shown between bases 40 and 60 in Figure 3). Strain E11 fits within one geno-group. Further, there is one single nucleotide polymorphism (SNP) representing a single base pair mutation at a specific SNP locus at position 119 in this alignment, at which a C/T transition is unique to this strain, indicating a likely clonal origin. *Karlodinium* strains from the Ebro Delta embayments from IRTA (Institut de Recerca i Tecnologia Agroalimentàries, Caldes de Montbui, Spain) (Table 1) were also analyzed by using two species-specific PCR assays. These PCR assays were not quantitative, but did allow for *Karlodinium* identification and differentiation of unknown samples by comparison of the unknowns to known samples previously identified by sequencing, and thereby matching the melt curve melting temperature (*T*m) and conformation of the melt curve profiles. Only two plankton samples collected from Alfacs Bay in 2000 had *K. armiger* present. All other samples strains were proven to contain *K. veneficum*, but different geno-groups of this species could be clearly differentiated.

Strain K10 from Alfacs Bay was previously identified, and had been isolated by the Institute of Marine Sciences (Barcelona, Spain), as *K. veneficum* strain ICMB 265 (GenBank: nucleotide sequence accession number jf906081); this was confirmed in the current assay. Similarly, strain CCMP2936 from Swann Keys, Selbyville, DE, USA was also in accordance with the molecular designation as *K. veneficum*.

### 2.2. New Karlotoxin Candidates

**Figure 4:**
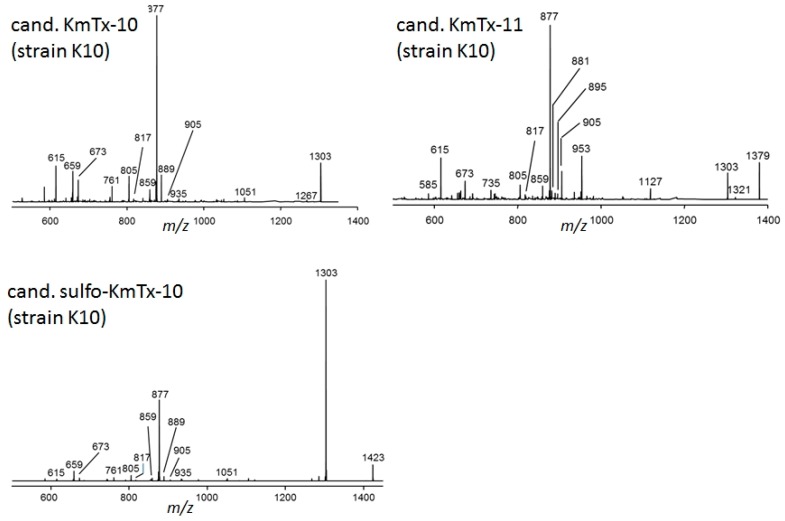
Collision induced dissociation (CID) spectra of cand. KmTx-10, cand. KmTx-11, and cand. sulfo-KmTx-10.

**Figure 5:**
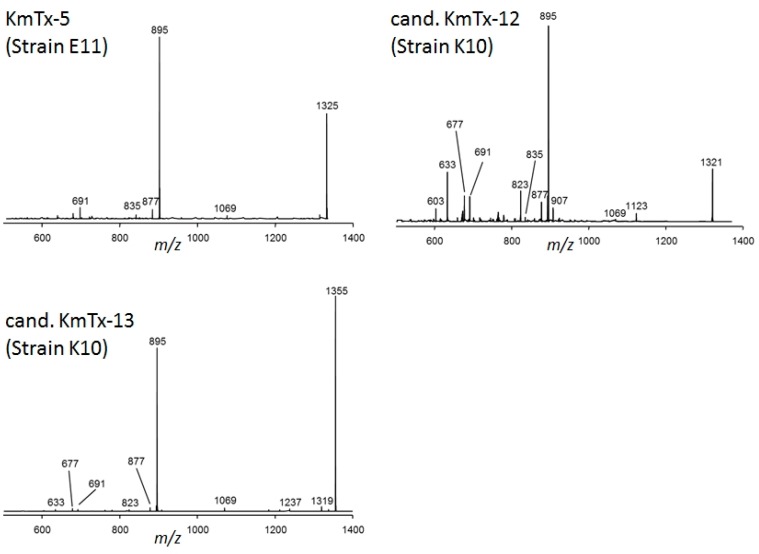
CID spectra of KmTx-5, cand. KmTx-12, and cand. KmTx-13.

Precursor ion experiments on the typical KmTx fragments *m*/*z* 877, 895, and 937 did not reveal other molecular masses in addition to those of KmTx-1 and KmTx-3 in strain CCMP2936. Collision induced dissociation (CID) spectra of these two compounds were consistent with KmTx-1 and -3, which have been previously identified in this strain [13]. Unexpectedly, the KmTx-2 reference solution proved not to be pure, but to contain 44-oxo-KmTx-2 as a minor impurity, which almost co-eluted with KmTx-2 under the chromatographic conditions in this method. Under the applied electrospray ionization (ESI) condition, no [M + H]^+^ ions of karlotoxins were formed, but sodium adducts instead. *K. veneficum* strain K10 isolated from Alfacs Bay, NW Mediterranean Sea, contained five putative karlotoxins with *m*/*z* 1303, 1321, 1355, 1379, and 1423, respectively. CID experiments on these compounds revealed spectra with typical fragments found for karlotoxins (Figure 4 and Figure 5); high resolution mass spectral experiments revealed their elemental compositions, and clearly indicated that all five compounds contained sulfur (Table 2). In continuation of the numbering of already described karlotoxins, these five new compounds were named as new candidate karlotoxins: cand. KmTx-10 (*m*/*z* 1303), cand. sulfo-KmTx-10 (*m*/*z* 1423), cand. KmTx-11 (*m*/*z* 1379), cand. KmTx-12 (*m*/*z* 1321), and cand. KmTx-13 (*m*/*z* 1355).

### 2.3. Karlotoxin Profiles

*Karlodinium veneficum* strain CCMP2936 originally isolated from the Atlantic US coast has been reported to produce KmTx-1 and KmTx-3 [14], and the presence of these two karlotoxins was confirmed. *K. veneficum* strain E11 isolated in Fangar Bay, Ebro Delta in 2012, produced KmTx-5 as the only known or putative karlotoxin. In contrast, *K. veneficum* strain K10 from adjacent Alfacs Bay showed a complex toxin profile comprising of the above mentioned five candidate karlotoxins, which have not been detected before (Table 3). A combination of the toxin profiles of strain E11 and K10 was found in four *K. veneficum* strains from Alfacs Bay, Ebro Delta, and consisted of the five novel candidate karlotoxins plus KmTx-5. In strain CCMP415, isolated from Norwegian coastal waters in 1985, none of the analyzed karlotoxins (Table 3) could be detected. The same applied to the two *K. armiger* strains isolated from Alfacs Bay in 2000.

### 2.4. Collision Induced Dissociation (CID) Spectra of Karlotoxins and Amphidinol-18 (AM-18)

**Figure 6:**
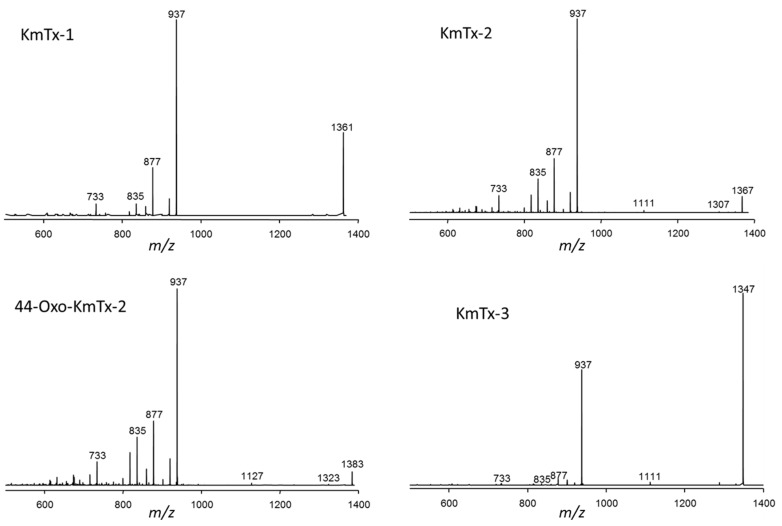
CID spectra of Kmtx-1, KmTx-2, 44-oxo-KmTx-2, and KmTx-3.

CID spectra of KmTx-1, KmTx-2, 44-oxo-KmTx-2, and KmTx-3 are very similar (as shown in Figure 6). The spectra of all four karlotoxins were characterized by the most abundant fragment *m*/*z* 937 that dominated all four spectra. The second most abundant fragment among all four spectra was *m*/*z* 877, which was least abundant in the spectrum of KmTx-3. In addition, all four spectra showed the fragments *m*/*z* 835 and *m*/*z* 733 at lower abundances. A second group of related spectra was formed by KmTx-5, cand. KmTx-12 and cand. KmTx-13 (Figure 5). These three spectra showed a fragmentation pattern similar to KmTx-1 to -3, but the dominant fragment was down-shifted about 42 Da to *m*/*z* 895. KmTx-5, cand. KmTx-12 and cand. KmTx-13 additionally share the lower abundance fragments *m*/*z* 877, *m*/*z* 691, *m*/*z* 677, and *m*/*z* 633.

A third group of CID spectra is formed by cand. KmTx-10, cand. KmTx-11, and cand. sulfo-KmTx-10 (Figure 4). These spectra are dominated by the fragment *m*/*z* 877, but also include *m*/*z* 1303 either as pseudo-molecular ion or as fragment. All three spectra share numerous lower mass fragments, such as *m*/*z* 859, 817, 805, 673, 659, and 615.

**Figure 7:**
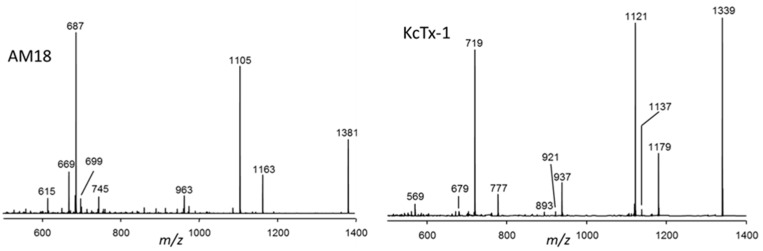
CID spectra of AM-18 and KcTx-1.

AM-18 and KcTx-1 showed more complex CID spectra with more abundant fragments over a wider mass range between *m*/*z* 600 and *m*/*z* 1200 (Figure 7).

### 2.5. Selected Reaction Monitoring (SRM) Method for the Detection of Karlotoxins

A LC-MS/MS method in the SRM mode was developed that is capable of resolving and identifying at least eleven defined and putative karlotoxins (KmTx-1, -2, -3, -5, 44-OH-KmTx-2, KcTx-1, cand. KmTx-10, cand. KmTx-12, cand. KmTx-13, cand. KmTx-11, and cand. sulfo-KmTx-10) and amphidinol-18 (Table 4). Two transitions for all compounds were selected for the SRM method (for details see Material and Methods section): the most intensive transition as the ion trace for quantitation and a second less abundant ion trace as qualitative control. This method was tested with different *Karlodinium* strains, and was proven to be able to detect the toxin profiles reported above.

### 2.6. Karlotoxin Cell Quota Estimations

The limit of detection defined as signal-to-noise ratio = 3 for KmTx-2 in the SRM mode was determined as 2.5 ng on-column, which corresponds to approximately 0.1 pg cell^−1^, based on a biomass of 10^6^ cells. Karlotoxin cell quotas of the analyzed strains were 2.5 pg cell^−1^, KmTx-3, and 7.9 pg cell^−1^ KmTx-1 for the strain CCMP2936 (Table 3) from Swann Bay, DE, USA. In general, karlotoxin cell quotas of the Mediterranean strains were one to two orders of magnitude lower, and quantitatively more variable than strains from elsewhere, ranging from 0.004 pg cell^−1^ cand. KmTx-13 in strain K0668, to 2.2 pg cell^−1^ cand. KmTx-12 in strain IRTA-SMM-12-17, expressed as KmTx-2 equivalents. Significantly, strain E11 from Fangar Bay of the Ebro Delta only produced KmTx-5, whereas all strains from Alfacs Bay, with exception of strain IRTA-SMM-12-07, produced less KmTx-5 on a cellular basis, but displayed a more complex toxin profile consisting of KmTx-5, cand. KmTx-10, cand. KmTx-12, cand. KmTx-13, cand. KmTx-11, and cand. sulfo-KmTx-10, with cand. KmTx-12 and cand. sulfo-KmTx-10 being the most abundant analogs (Table 3).

### 2.7. Hemolytic Activity of Cand. Sulfo-KmTx-10

**Figure 8:**
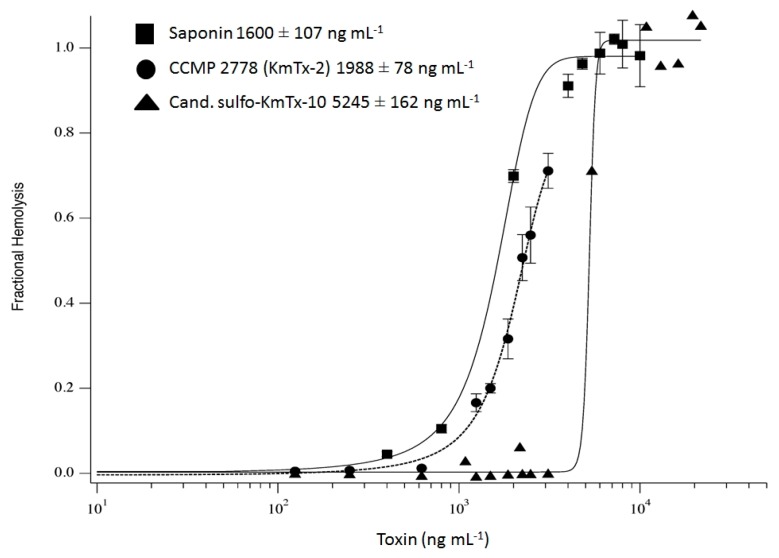
Comparative hemolytic potency for cand. sulfo-KmTx-10 (filled triangles) to KmTx-2 (filled circles) and saponin (filled squares) using Gilthead seabream (*Sparus aurata*) erythrocytes. Note the logarithmic scale for toxin amount. The fitted line is based on the Hill equation, and the HD_50_ (toxin concentration giving 0.5 fractional hemolysis) estimates for these curves are presented in the legend to the figure.

Only cand. sulfo-KmTx-10 was purified (procedure in Supplemental [15]) in sufficient quantity and quality to perform quantitative hemolytic activity assays. The new cand. sulfo-KmTx-10 exhibits hemolytic activity against gilthead seabream (*Sparus aurata*) erythrocytes (Figure 8), with an 50% inhibitory concentration (IC_50_) of 5245 ± 162 ng mL^−1^, which is much higher (i.e., less potent) compared to pure KmTx-2 with an IC_50_ of 1988 ± 78 ng mL^−1^. This is consistent with the finding that sulfatation of karlotoxin reduces its hemolytic activity [14].

## 3. Discussion

### 3.1. Liquid Chromatography-Tandem Mass Spectrometry LC-MS/MS Method

The LC-MS/MS method developed in this study is based on the SRM mode, and allows for the qualitative detection and quantification of the respective karlotoxins and one related amphidinol analog. The method is based upon two transitions per analyte: the transition of the [M + Na]^+^ pseudo-molecular ion (as sodium adducts were the most abundant pseudo-molecular ions, and [M + H]^+^ ions were absent) to the most abundant fragment. In contrast to other dinoflagellate polyketides toxins, such as okadaic acid/dinophysistoxins, pectenotoxins, azaspiracids, or spiroimines that are characterized by several water losses from their pseudo-molecular ions, the most abundant fragments formed by karlotoxins result from a cleavage of the C–C bond between C41 and C42 (Figure 2) [14]. This transition is used for quantification. As water losses in CID spectra of karlotoxins are barely observed, the second most abundant transition of each compound used as quality control also results from a C–C cleavage. Two transitions per analyte based on carbon skeleton cleavages guarantee a highly specific detection of karlotoxins. Furthermore, the retention time window of karlotoxins in this method, ranging from 9.1 to 10.3 min (Table 4), is rather narrow, and additionally facilitates the recognition of this toxin class. The current method comprises and confirms only the selected available eleven karlotoxins and one amphidinol, but it can be easily extended by addition of new transitions of other karlotoxins and amphidinols of interest, if their CID spectra or fragmentation patterns are known. Fragmentation of karlotoxins and amphidinols differs from most other polyketide marine phycotoxins, and requires different ionization parameters in the ion source of the mass spectrometer, such as high fluxes of nebulizer and auxiliary gases, in addition to high declustering potential and collision energy due to the stability of sodium adducts. These parameters complicate the integration of karlotoxins and amphidinols into other multi-methods for the screening of marine phycotoxins.

### 3.2. Quantitation and Method Potential and Limitation

Quantification of phycotoxins depends on the availability of calibration standards. Due to their high molecular weight and complex chemical structures, there are no stable isotope labeled phycotoxins available that could be used as internal standards for toxin quantification, which would be the most accurate quantification method as it also accounts for possible matrix effects. As internal calibration is not possible, calibration of phycotoxin containing samples is usually done by external calibrations based upon analyte solutions with known concentrations. To the best of our knowledge, there are no commercially available karlotoxin standards. For this reason, all karlotoxins of this study were determined with respect to a calibration solution of KmTx-2 that was previously purified from a *K. veneficum* culture [15], and all values are given as KmTx-2 equivalents, assuming a similar molecular response between KmTx-2 and the karlotoxins of this study. This assumption is justified by the fact that the fragments used for quantitation result from the same cleavage pattern in most cases. The latter is not true for KcTx-1, AM-18, and cand. sulfo-KmTx-10, that show a slightly different fragmentation pattern. For this reason, all values given as KmTx-2 equivalents are not accurate determinations, but rather semi-quantitative estimates. In many cases, where analytical standards are not available, estimates can be sufficient for many scientific purposes, such as determination of toxin absence/presence, distribution in field samples or variation in profile, and quantitative composition in lab experiments.

Karlotoxins and amphidinols, like other relatively high molecular weight polyketide phycotoxins, such as yessotoxins, brevotoxins, paly-/ovatoxins, and ciguatoxins, do not easily ionize in the ion sources of mass spectrometers. In general, this results in a lower detection sensitivity in comparison to other phycotoxins. The limit of detection (LOD) for KmTx-2 was determined as 2.5 ng on-column, which corresponds to 0.1 pg cell^−1^ on a cellular basis, i.e., with 10^6^ extracted cells. This LOD falls in the range of estimated karlotoxin cell quotas (Table 3), and can be reduced by the use of more biomass in the case of available cultures. The fact that one million cells are needed to reach the LOD of this method, assuming an average toxin cell quota of 0.1 pg cell^−1^, argues against the possibility of detecting karlotoxins in field samples with low or background concentrations of *Karlodinium* cells. For example, in Fangar Bay, no high cell abundances were detected before 2010 [18], and in Alfacs Bay, *Karlodinium* concentrations are generally below 10,000 cell L^−1^. Only on three occasions during the period between 1990 and 2009 did the *Karlodinium* cell concentrations in Alfacs Bay exceed the alert concentration of 2 × 10^5^ cells L^−1^ set by the monitoring program of water quality for shellfish growing areas in Catalonia [19], reaching maximum levels of almost 10^6^ cells L^−1^ [20]. Accordingly, no karlotoxins were detected by LC-MS/MS in field plankton samples taken during May to July in 2010 and 2011 from Alfacs and Fangar Bay [17], especially taking into account that the Mediterranean strains mainly produced karlotoxins, which were unknown at that time. On the other hand, sampling of karlotoxins by cumulative passive samplers, such as solid phase adsorption toxin tracking (SPATT) resins [21], is expected to efficiently sample karlotoxins. Most current resins used for SPATTs selectively adsorb lipophilic and high molecular weight organic compounds [22], and cumulatively retain these compounds during time series field deployment, to above the respective LOD. The testing of SPATTs for field sampling of karlotoxins has not been addressed in this study, but is pending for future work.

### 3.3. Characteristics of New Karlotoxins

It is noteworthy that all five novel putative karlotoxins of the Mediterranean strains of *K. veneficum* contain sulfur, and are most likely sulfated karlotoxin variants (Table 2). This is quite obvious for cand. sulfo-KmTx-10, which is a sulfated form of cand. KmTx-10 that eliminates NaHSO_4_ (120 Da) to form *m*/*z* 1303 as most abundant fragment (Figure 4). Sulfatation has been previously observed for other lipophilic polyketides, such as okadaic acid and dinophysistoxin-1, which are potent PP1 and PP2A protein phosphatase inhibitors, and it has been suggested that these less toxic sulfated forms of the marine biotoxins might be biosynthetic precursors to prevent a self-intoxication of the producing organism [23,24,25]. But amphidinols structurally closely related to karlotoxins (Figure 2), were also reported to be sulfated [26]. According to their CID spectra, the five new putative karlotoxins can be classified into two groups: one group of karlotoxins forming *m*/*z* 895 as the most abundant fragment with cand. KmTx-12 and cand. KmTx-13 (Figure 5), and a second group with cand. KmTx-10, cand. KmTx-11, and cand. sulfo-KmTx-10, with *m*/*z* 877 as most abundant fragment (Figure 4). The two compounds with the fragment *m*/*z* 895 share this fragment with KmTx-5, suggesting that cand. KmTx-12 and cand. KmTx-13 are close structural variants of KmTx-5. The fact that KmTx-5, cand. KmTx-12, and cand. KmTx-13 yield fragment *m*/*z* 895, which is formed by the cleavage between C41 and C42 (Figure 2), and have some other smaller fragments in common, is clear evidence that all three compounds are conserved in the C1–C41 region, and that all modifications have to be located in the more lipophilic part of the molecule >C42. In contrast, the most abundant fragment of the second group comprising cand. KmTx-10, cand. KmTx-11 and cand. sulfo-KmTx-10 is *m*/*z* 877 (Figure 4), which is also formed by KmTx-5, cand. KmTx-12, and cand. KmTx-13 (Figure 5), by a water loss of fragment *m*/*z* 895. Fragment *m*/*z* 895, even though not being the base peak, is also visible in the CID spectrum of cand. KmTx-11, highlighting its similarity to KmTx-5, cand. KmTx-12, and cand. KmTx-13. The shift of *m*/*z* 895 to the 18 Da lower fragment *m*/*z* 877 in cand. KmTx-10, cand. KmTx-11, and cand. sulfo-KmTx-10, is probably due to change in substitution or chemistry in the C41–C42 region of the molecules, that favors an additional water loss resulting in *m*/*z* 877 over the intact fragment *m*/*z* 895 in cand. KmTx-11, and complete suppression of *m*/*z* 895 in cand. KmTx-10 and cand. sulfo-KmTx-10. These chemical features can only be verified by nuclear magnetic resonance (NMR) spectroscopy of purified compounds in sufficiently high amounts, which were not available for this study. Nevertheless, the similarity of the CID spectra of the five novel compounds are strong evidence that these new compounds belong to the class of karlotoxins.

### 3.4. Structural Variations of Karlotoxins, Similarities to Amphidinols and Function

The list of described karlotoxins now contains more than 20 variants, and further exploration will likely reveal a much higher number of karlotoxin variants than currently known. The karlotoxin profile of strain E11, consisting only of KmTx-5, initially described in a strain from Plymouth Sound, England [2], compared with that of K10, comprising five novel karlotoxins (Table 3), indicates that there are at least two *K. veneficum* toxin phenotypes in the northwestern Mediterranean, within a narrow geographical range from embayments of the Ebro Delta. Other cultures from the Ebro Delta show a combination of both toxin profiles, probably reflecting the fact that these *K. veneficum* cultures represent mixtures of different geno-groups, and thus, may display a superposition of different toxin profiles.

High structural variation also has been observed within other groups of marine phycotoxins, such as yessotoxins [27], spiroimines [28], and azaspiracids [29], and this is not surprising given the high molecular weight of these polyketides. Even though the ecological function of many groups of phycotoxins are not known, it can be assumed that chemical variability does not dramatically affect the function of these molecules. The situation in the case of karlotoxins is somewhat clearer, as these compounds have been shown to have lytic activity by non-selective permeabilization of plasma membranes, leading to osmotic cell lysis [30]. This effect has been linked to ichthyotoxicity of *K. veneficum* [31]. It is reasonable to assume that the fish killing effect is not the underlying ecological function, but rather a side effect, as it has been shown that cell lysis caused by other marine dinoflagellates, such as *Alexandrium* or *Protoceratium*, has a protective effect against their protistan grazers, and may even reverse trophic relationships [32,33]. The fact that *Alexandrium* lytic compounds, which unlike karlotoxins have not been fully identified, also act via unspecific permeabilization of membranes [34], suggests that these large molecules with amphoteric properties [35] share structural principles with karlotoxins. It has been proven that the production of karlotoxins in *Karlodinium* acts as a defense against protistan predation [11] and parasitism [36]. This hypothesis is supported by the fact that karlotoxins (unlike other phycotoxins) are excreted by *Karlodinium* [4,8], and their amphoteric characteristic enables a certain water solubility provided by the more hydrophilic, highly hydroxylated part of the molecules necessary for any ecological effect, and the lipophilic part is capable of eliciting the lytic effect on membranes of other marine organisms. This structural feature is shared with amphidinols that are structurally closely related to karlotoxins, and also consist of a highly hydroxylated part and a more lipophilic end (Figure 2). This leads to the conclusion that the functional principle of cell lysis of competitors or grazers is not constrained to *Karlodinium*, but constitutes a rather general chemical weapon in the marine battle field.

There is also the hypothesis that karlotoxins evolved for prey capture [10]. If strains of *K. veneficum* do not produce karlotoxin, they are unable to capture and ingest cryptophytes [10]. Many strains isolated from the NW Mediterranean exhibit variable mixotrophy [37], which could be correlated with toxin quota and congener.

Among the analyzed strains, CCMP415 (accession number AJ557026 in Figure 3) from the Norwegian coast did not contain any of the karlotoxins detected by the SRM method developed in this study. At this stage, it is difficult to interpret this negative result, as there are three alternative explanations; the strain may (1) be toxigenic, but produce a different toxin profile, including novel karlotoxins and/or those that are not included in the method; (2) have lost its toxicity, as it was isolated into unialgal culture more than 30 years ago; or (3) be non-toxic, and not produce any karlotoxins. Mixed populations of toxic and non-toxic dinoflagellates within one species have been observed in *Alexandrium* [38].

## 4. Materials and Methods

### 4.1. Isolation and Culturing of Karlodinium Strains

Isolation of *K. veneficum* strains from the Ebro Delta embayments, Alfacs (40.6200838° N, 0.6581678° E) and Fangar (40.7787678° N, 0.7492338° E), in the northwest Mediterranean Sea (Table 1) was conducted from filtered (20 µm Nitex gauze) 1 L water samples. Aliquots of 20 µL of the preliminary isolates were transferred into 200 µL 1/10 strength K-medium in individual wells of 96 well tissue culture plates (TPP, Trasadingen, Switzerland). Plates were incubated at 15 °C under a photon flux density of approximately 50 µmol m^−2^ s^−1^ on a 16:8 h light/dark photocycle in a controlled environment growth chamber (Model MIR 252, Sanyo Biomedical, Wood Dale, IL, USA). The plates were regularly inspected for the presence of motile cells under a stereomicroscope (Olympus SZH-ILLD; Olympus, Hamburg, Germany) with dark field illumination. From each well containing rapidly growing *Karlodinium*-like cells, single cells were isolated under a stereomicroscope (M5A, Wild, Heerbrugg, Switzerland) by micropipette, and transferred to fresh wells with the same medium and incubated as described above. In some cases, cloning of individual cells was not successful, but the culture was maintained as a mixture of *Karlodinium* spp. One clonal isolate, provisionally named E11, was retained for further scale-up culture in plastic culture flasks under the defined culture conditions described below. Observation and microscopic documentation of live cells (Figure 1) was carried out with a compound microscope (Axiovert 2, Zeiss, Göttingen, Germany) equipped with differential interference contrast optics.

For toxin analysis, strain E11 (Table 1) was grown in three replicate 65 mL culture flasks at 15 °C under a photon flux density of 60 µmol m^−2^ s^−1^ on a 16:8 h light/dark photocycle. Growth was determined from 1 mL samples, collected daily by counting cells in a Sedgewick-Rafter chamber after fixation with Lugol’s iodine solution, and counting of >800 cells under an inverted microscope. Cells were harvested in exponential growth phase, when the culture cell density reached approximately 3 × 10^4^ cells mL^−1^.

The *Karlodinium* cultures grown at IRTA (CCMP415, IRTA-SMM-12-23, IRTA-SMM-12-01, K0668, IRTA-SMM-12-17, IRTA-SMM-12-03, IRTA-SMM-12-07, *K. armiger* 1 and *K. armiger* 2; Table 1) were maintained at 21 °C under a 12:12 h light/dark cycle, and ES growth medium [39] in glass bottles, and were harvested at the exponential phase. An aliquot of 50 mL was preserved in Lugol’s iodine solution for determination of cell abundance by microscopic counting. Cell density was determined by settling Lugol’s iodine-fixed samples, and counting of >100 cells under an inverted microscope.

### 4.2. Molecular Techniques

#### 4.2.1. DNA Extraction for *Karlodinium* Species Assignment

For DNA extraction of *Karlodinium* E11, and other cultured strains, 50 mL of an exponentially growing culture at a density of 9.5 × 10^4^ cells mL^−1^ (determined by microscopic counts as described above) was harvested by centrifugation (Eppendorf 5810R, Hamburg, Germany; 3220× *g* for 10 min). The pellet was transferred to a microtube, again centrifuged (Eppendorf 5415, 16,000× *g*, 5 min), and stored frozen at −80 °C until DNA extraction. Genomic DNA was extracted with a DNeasy Plant Mini Kit (Qiagen, Hamburg, Germany). In brief, pellets were re-suspended in 400 µL pre-heated (65 °C) AP1 lysis buffer (DNeasy; Qiagen), and transferred to 2 mL tubes with 300–500 µL acid washed glass beads (80–200 µm) (Sigma-Aldrich, Munich, Germany), in which cells were disrupted with a Fast Prep tissue homogenizer (2 × 20 s at 6.5 speed). Four microliters of RNase were transferred into tubes, and DNA was extracted following the manufacturer´s protocol. Nucleic acid extractions from seawater samples were prepared by a primary centrifugation step to collect all solids in a pellet; then, DNA was extracted from the pellets, following previously published protocols [40]. Concentration of the resulting DNA samples was then determined with Nano Drop, and 20 ng DNA was checked for degradation on a 1% agarose gel.

#### 4.2.2. Polymerase Chain Reaction Template Amplification of *Karlodinium* spp. from the Mediterranean

Polymerase chain reaction (PCR) of the internal transcribed spacer (ITS) and large subunit (LSU) region of the 28 S rDNA gene, was achieved with ITS primers ITSa and ITSb, and LSU primers DirF and Dir2CR, respectively. The details of the respective primers are as follows: *LSU-Primers* [41] DIRF: 5′-ACC CGC TGA ATT TAA GCA TA-3′ (forward Pprimer) D2CR: 5′-CCT TGG TCC GTG TTT CAA GA-3′ (reverse primer) *ITS-Primers* ITS a: 5′-CCA AGC TTC TAG ATC GTA ACA AGG (ACT)TC CGT AGG T-3′ (forward primer) ITS b: 5′-CCT GCA GTC GAC A(GT)A TGC TTA A(AG)T TCA GC(AC) GG-3′ (reverse primer). The PCR reactions were carried out with the following master mix: 16.3 µL deionized water, 2.0 µL 10× HotMaster Taq buffer, 0.2 µL 10 µM forward primer, 0.2 µL 10 µM reverse primer, 0.2 µL 10 µM dNTPs, 0.1 µL Taq polymerase and 1 µL DNA (10 ng µL^−1^). For PCR of the ITS region, the thermal cycling of the mix was set up with an initial denaturation step at 94 °C for 4 min, followed by 10 cycles at 94 °C for 50 s, 58 °C for 40 s, and at 70 °C for 1 min, thereafter followed by 30 cycles of 94 °C for 45 s, then 45 s at 50 °C and 1 min at 70 °C, and a final elongation step at 70 °C for 5 min. For PCR amplification of the LSU, this treatment was modified as follows: one cycle of denaturation for 2 min at 94 °C, 30 cycles at 94 °C for 30 s, then at 55 °C for 30 s, and at 65 °C for 2 min, followed by a final elongation at 65 °C for 10 min.

*Karlodinium*-specific primers [42] were also utilized for differentiating amplicons obtained from DNA extracts of bloom samples (seawater DNA extracts) by comparing melt curve profiles of previously sequenced strains to the melt curves obtained from seawater extracts (Table 1). Amplifications were performed in an ABI 7300 (Life Technologies, Carlsbad, CA, USA) in a 20 µL volume containing 0.5 µM of each primer and 1× concentration of SYBR Green reaction mix (Ref# 4364344; Life Technologies).

#### 4.2.3. Amplicon Preparation and Sequencing

Success of the PCR amplification for LSU and ITS was confirmed on 1% agarose gel. The PCR products were then cleaned up with a MinElute PCR purification kit (Qiagen). The sequencing reactions were carried out with 1 µL purified PCR product, 1.5 µL Big Dye buffer, 1 µL Big Dye, 6.5 µL water, and 1 µL forward primer (1 µM) for the forward reaction, and 1 µL reverse primer (1 µM) for the reverse reaction, respectively. Sequencing was followed by cleaning of the products with CleanSeq–Beads (Beckmann Coulter, Brea, CA, USA) following manufacturer´s protocol, with elution in 0.1 mM EDTA. Sequencing was carried out with an ABI Prism sequencer. Sequencing reactions were carried out at 96 °C for 1 min, followed by 25 cycles at 96 °C for 10 s, at 50 °C for 5 s, and at 60 °C for 4 min.

#### 4.2.4. DNA Sequence Analysis and Molecular Taxonomic Assignment

Raw sequence data were analyzed (CLC genomic workbench v 6.0), and forward and reverse sequences aligned to construct a consensus sequence. Reads matching to known *K. veneficum* sequences were exported as fasta files. Fasta files were then aligned in BioEdit to confirm their association to *K. veneficum* and the different geno-groups within the species.

### 4.3. Chemical Analysis

#### 4.3.1. Harvest of *Karlodinium* Cells and Extraction of Karlotoxins

A biomass of *Karlodinium* strains K10 and CCMP2936, equivalent to 150 × 10^6^ and 128 × 10^6^ cells, respectively, was harvested by filtration (Whatman^®^ glass microfiber filters, Grade GF/F, GE Healthcare Life Sciences, Piscataway, NJ, USA) under low vacuum (<100 mm Hg). The filters with retained cells were then extracted sequentially with methanol (50 mL) and acetone (50 mL). The extraction process was ultrasonication-aided by an ultrasonic probe-type device (UP200S, Hielscher Ultrasonics™; 200 W, 24 kHz, 4 min, Hielscher, Teltow, Germany). The sonication power was 50% of full power, and the pulse control was set at 0.5. The sonicated suspensions were centrifuged (4000× *g*, 10 min, 10 °C), the methanolic and acetonic supernatants were recovered and filtered (0.22 µm pore size membrane filter) to remove debris. The methanolic and acetonic extracts of each strain were combined and dried under a N_2_ stream.

All strains grown at IRTA were harvested by filtration (Whatman GF/F) under low vacuum. The filters were maintained in 80% methanol at −20 °C until analysis.

A total of 150 mL of the strain E11 culture (corresponding to 5.1 × 10^6^ cells) were harvested by centrifugation (Eppendorf 5810R) at 3220× *g* for 10 min. All pellets were combined in a microtube, again centrifuged (Eppendorf 5415, 16,000× *g*, 5 min), and stored frozen (−20 °C) until further extraction. Archived cell pellets were later suspended in 0.5 mL methanol, and homogenized by ultrasonication (Sonoplus HP 2070, Bandelin, Berlin, Germany) for 60 s (for 70 cycles at 90% power). The homogenate was centrifuged for 3 min at 5900× *g* (Eppendorf 5415R) and the supernatant stored at −20 °C until analysis.

#### 4.3.2. Solid Phase Extraction (SPE) Clean-Up of *Karlodinium* Extracts

Methanolic extracts of all strains were diluted with deionized water to a final 20% methanol concentration. SPE cartridges (6 mL, LC-18 Supelclean, Sigma-Aldrich, Deisenhofen, Germany) were conditioned with 2 mL methanol, and subsequently equilibrated with 2 mL 50% aqueous methanol and 2 mL deionized water. Samples were loaded onto the equilibrated SPE cartridges and cartridges washed with 2 mL deionized water and 50% aqueous methanol. Karlotoxins were eluted with 2 mL 80% methanol. Samples were dried in a gentle N_2_ stream, and dissolved in 500 µL methanol. Methanolic extracts were passed through a spin-filter (0.45 µm, Ultrafree, Millipore, Eschborn, Germany) by centrifugation for 2 min at 3300× *g* prior to karlotoxin analysis.

#### 4.3.3. LC-MS/MS Precursor Ion Spectra

Concentrated extracts of *Karlodinium* strains K10, CCMP2936, the *K. veneficum* isolate E11 from Fangar Bay, and the KmTx-2 standard solution were run in three precursor ion experiments with typical KmTx fragments, to determine the possible presence of other KmTx-related compounds. Precursor ion experiments were performed on a triple quadrupole mass spectrometer (API 4000 QTrap, AB Sciex, Darmstadt, Germany) equipped with a Turbo lon Spray interface, coupled to a liquid chromatograph (model 1100, Agilent, Waldbronn, Germany). The liquid chromatograph includes a solvent reservoir, in-line degasser (G1379A), binary pump (G1311A), refrigerated autosampler (G1329A/G1330B), and temperature-controlled column oven (G1316A). Separation of lipophilic toxins was performed after injection of 10 μL sample by reversed-phase chromatography on a C8 phase. The analytical column (50 × 2 mm) was packed with 3 μm Hypersil BDS 120 Å (Phenomenex, Aschaffenburg, Germany) and maintained at 20 °C. The flow rate was 0.2 mL min^−1^, and gradient elution was performed with two eluants, where eluant A was water and eluent B was acetonitrile/water (95:5 *v*/*v*), both containing 2.0 mM ammonium formate and 50 mM formic acid. Initial conditions were 8 min column equilibration with 5% B, followed by a linear gradient to 100% B in 8 min, and isocratic elution until 15 min with 100% B. The system was then returned to initial conditions (total run time: 23 min). Precursors of the fragments *m*/*z* 877, *m*/*z* 895, and *m*/*z* 937 were scanned in the positive ion mode from *m*/*z* 1150 to 1500. Mass spectrometric parameters are detailed in Table 5.

#### 4.3.4. LC-MS/MS Collision Induced Dissociation (CID) Spectra

Collision induced dissociation (CID) product ion spectra were recorded for the [M + Na]^+^ adduct ions of all available compounds: amphidinol-18 (AM-18), karlotoxin-1 (KmTx-1), KmTx-2, 44-oxo-KmTx-2, KmTx-3, KmTx-5, *Karlodinium conicum* toxin-1 (KcTx-1), cand. KmTx-10, cand. KmTx-11, cand. KmTx-12, cand. KmTx-13, and cand. sulfo-KmTx-10. CID spectra were recorded on the same instrument in the enhanced product ion (EPI) modus in the mass range from *m*/*z* 500 to 1430, and in positive ionization and unit resolution mode. Mass spectrometric parameters are detailed in Table 5.

#### 4.3.5. LC-MS/MS Selected Reaction Monitoring (SRM)

LC conditions were as described above. The two most abundant fragments of each compound were selected to develop a selected reaction monitoring (SRM) method. SRM experiments were performed on the same instrument as above in the positive ion mode. The transitions used, and their respective toxins, are listed in Table 4. Two transitions for each compound were selected in order to increase specificity of this method. Dwell times of 10 ms were used for each transition. Mass spectrometric parameters are detailed in Table 5.

Samples were calibrated against an external KmTx-2 standard solution (23 ng µL^−1^), and all karlotoxin values were expressed as KmTx-2 equivalents, assuming a similar molecular response among all karlotoxin variants.

#### 4.3.6. High Resolution Mass Spectroscopy

One milliliter of the *K. veneficum* K10 methanolic raw extract was diluted with 4 mL water to achieve a methanol concentration not higher than 20%. This solution was purified by SPE, as described above. The eluate was dried in a N_2_ stream, and dissolved in 600 µL methanol.

High resolution mass spectra were acquired with a Solarix XR Fourier transform ion cyclotron resonance mass spectrometer (FT-ICR-MS; Bruker Daltonik GmbH, Bremen, Germany) equipped with a 12 T refrigerated actively shielded superconducting magnet (Bruker Biospin, Wissembourg, France), a dual ion source, and Paracell analyzer cell [43]. The samples were ionized by electrospray ionization in positive ion mode. Sample solutions were continuously infused using a syringe at a flow rate of 2 µL min^−1^. The detection mass range was set to *m*/*z* 150−3000. Ion accumulation time for each scan was set to 0.05 s. Several scans were added for the final mass spectrum. Data sets were acquired with 8 MW data points, yielding a resolving power of 900,000 at *m*/*z* 400. Spectra were zero-filled to process size of 16 M data points before sine apodization. Mass spectra were calibrated with NaTFA clusters using a linear calibration. A 0.1 mg mL^−1^ solution of NaTFA in 50% methanol was used to generate the clusters. Ion accumulation time was set to several seconds for MS/MS experiments for improved S/N of the fragment mass peaks. The quadrupole isolation window was set to between 3 and 6 Da, and collision energy to 50 eV.

### 4.4. Hemolytic Assay

Gilthead seabream (*Sparus aurata*) erythrocytes were extracted from the caudal vein and the blood washed three times with three volumes (per mL) of buffer of packed erythrocytes, using the incubation buffer described below (minus CaCl_2_). Following the third wash, the cells were resuspended in 3 mL of buffer per mL of packed cells. The resuspended cells were diluted 1:20 in buffer (pH 7.5; 150 mM NaCl, 3.2 mM KCl, 1.25 mM MgSO_4_, 12.2 mM Tris Base, and 3.75 mM CaCl_2_) and 200 µL of diluted, washed erythrocytes were incubated for 1 h at 25 °C in the presence of purified cand. sulfo-KmTx-10, serially diluted in methanol for testing. After incubation, remaining intact erythrocytes were pelleted by centrifugation, and the optical density (OD) at 540 nm was read with a Molecular Devices Vmax (Sunnyvale, CA, USA) microtiter plate spectrophotometer. Saponin (0 to 20 µg) from Quillaja bark (Sigma-Aldrich, S-4521, St Louis, MO, USA) was used as a positive control. The dose response curves [Fractional Hemolysis vs. log (µg mL^−1^ of karlotoxin)] were fitted to the Hill equation with a nonlinear regression model (Igor 6.02, Wavemetrics, Lake Oswego, OR, USA):

fractional hemolysis = base hemolysis + (maximal hemolysis − base hemolysis)/[1+ (toxin giving 50% hemolysis) ^rate of hemolysis^ (1)

In all cases, convergence to defined parameters was observed.

Known aliquots of the purified toxins were placed onto a tared aluminum weigh boat in triplicate, dried at 60 °C overnight, and weighed the following day on a microgram balance (Mettler UMT2, Columbus, OH, USA). Cand. sulfo-KmTx-10 was also serially diluted in methanol for testing. Known dilutions of cand. sulfo-KmTx-10 were then mixed with water (25 µL toxin + 50 µL water), and 5, 10, and 50 µL injections of each sample were run in triplicate to determine the UV and MS response factors of the material tested.

## 5. Conclusions

This work presents a SRM method for the qualitative and quantitative determination of targeted karlotoxins and amphidinol-18 with a detection limit of 2.5 ng KmTx-2 on-column. The method can be expanded by transitions of further karlotoxins and amphidinols for specific needs. During experiments with several *Karlodinium veneficum* strains from the western Mediterranean Sea, five novel putative karlotoxins were found and integrated into the SRM method. Toxin analysis of available *K. veneficum* strains showed a high chemical variability of karlotoxins within a field population, but apparently also among geographically distinct populations.

All of prior work suggests that toxin production in *K. veneficum* strains is genetically determined, but cellular toxin quotas are highly variable [9]. The majority of strains show a karlotoxin from detection limit to the pg cell^−1^ range, although nontoxic strains also have been isolated. These results suggest that under certain growth conditions, different strains will respond differently in terms of quantitative toxin production, but expressed toxin patterns are stable during the growth cycle.

## Figures and Tables

**Table 1:** Strain designations, number of harvested cells for toxin analysis, molecular species identification by quantitative PCR (qPCR) of rDNA gene, and geographical origin of *Karlodinium* strains.

Strain #	Harvested Cells	Species Identification	Geographical Origin
E11	5.10 × 10^6^	*K. veneficum*	Fangar Bay, Ebro Delta (2012)
K10	1.79 × 10^8^	*K. veneficum*	Alfacs Bay, Ebro Delta (2007)
CCMP2936	1.28 × 10^8^	*K. veneficum*	Swann Keys, DE, USA (2006)
CCMP415	1.67 × 10^9^	*K. veneficum*	Norway (1985)
IRTA-SMM-12-23	6.35 × 10^7^	*K. veneficum*	Alfacs Bay, Ebro Delta (2012)
IRTA-SMM-12-01	8.36 × 10^8^	*K. veneficum*	Alfacs Bay, Ebro Delta (2012)
K0668	2.37 × 10^8^	*K. veneficum*	Alfacs Bay, Ebro Delta (2002)
IRTA-SMM-12-17	1.20 × 10^8^	*K. veneficum*	Alfacs Bay, Ebro Delta (2012)
IRTA-SMM-12-03	6.63 × 10^8^	*K. veneficum*	Alfacs Bay, Ebro Delta (2012)
IRTA-SMM-12-07	1.35 × 10^8^	*K. veneficum*	Alfacs Bay, Ebro Delta (2012)
*K. armiger* 1 *	6.24 × 10^7^	*K. armiger*	Alfacs Bay, Ebro Delta (2000)
*K. armiger* 2 *	4.45 × 10^7^	*K. armiger*	Alfacs Bay, Ebro Delta (2000)

* *K. armiger* strains were not tested for karmitoxin.

**Table 2:** Exact masses, elemental composition, and error between theoretical and empirical masses.

Toxin	*m*/*z* Observed	Elemental Composition	±ppm
cand. KmTx-10	1303.733892	C_62_H_117_ClNaO_22_S	0.08
cand. KmTx-11	1379.748807	C_64_H_121_ClNaO_25_S	0.8
cand. sulfo-KmTx-10	1423.682504	C_62_H_118_ClNa_2_O_26_S_2_	0.4
cand. KmTx-12	1321.742175	C_62_H_119_ClNaO_23_S	1.0
cand. KmTx-13	1355.749870	C_62_H_121_ClNaO_25_S	0.2

**Table 3:** Karlotoxin cell quotas (pg cell^−1^) expressed as KmTx-2 equivalents of twelve *Karlodinium* strains investigated in this study (- means not detected).

Strain	KmTx-1	KmTx-3	KmTx-5	Cand. KmTx-10	Cand. KmTx-12	Cand. KmTx-13	Cand. KmTx-11	Cand. Sulfo-KmTx-10
E11	-	-	0.21	-	-	-	-	-
K10	-	-	-	0.03	0.58	0.002	0.003	0.07
CCMP2936	7.9	2.5	-	-	-	-	-	-
CCMP415	-	-	-	-	-	-	-	-
IRTA-SMM-12-23	-	-	0.05	0.08	1.2	0.10	0.02	0.5
IRTA-SMM-12-01	-	-	-	-	-	-	-	-
K0668	-	-	0.004	0.006	0.16	0.003	0.009	0.09
IRTA-SMM-12-17	-	-	0.08	0.09	2.2	0.40	0.15	0.64
IRTA-SMM-12-03	-	-	-	-	-	-	-	-
IRTA-SMM-12-07	-	-	0.57	0.006	0.28	0.01	0.02	0.03
*K. armiger* 1	-	-	-	-	-	-	-	-
*K. armiger* 2	-	-	-	-	-	-	-	-

**Table 4:** Names, Q1 and Q3 masses, retention times, and molecular weights of the toxins detected by this method. Transitions (Q1 > Q3) in bold are the most abundant.

Toxin	Q1 Mass (*m*/*z*)	Q3 Mass (*m*/*z*)	Retention Time (min)	Molecular Weight (g mol^−1^)
AM-18	1381.8 **1381.8**	1105.6 **687.6**	9.9	1358.8
KcTx-1	**1339.8** 1339.8	**1121.7** 719.6	9.7	1316.8
KmTx-1	**1361.8** 1361.8	**937.6** 877.6	10.2	1338.8
KmTx-2	**1367.8** 1367.8	**937.6** 877.6	10.0	1344.8
44-oxo-KmTx-2	**1383.8** 1383.8	**937.6** 877.6	10.0	1360.8
KmTx-3	**1347.8** 1347.8	**937.6** 877.6	10.3	1324.8
KmTx-5	**1325.8** 1325.8	**895.6** 691.5	10.1	1302.8
cand. KmTx-10	**1303.8** 1303.8	**877.6** 615.4	9.6	1280.7
cand. KmTx-12	**1321.8** 1321.8	**895.6** 633.4	9.8	1298.7
cand. KmTx-13	**1355.8** 1355.8	**895.7** 677.5	9.1	1332.7
cand. KmTx-11	**1379.8** 1379.8	**877.6** 615.4	9.9	1356.7
cand. sulfo-KmTx-10	1423.8 **1423.8**	1303.8 **877.6**	9.8	1400.7

**Table 5:** Mass spectrometric parameters for the detection of karlotoxins in the precursor ion mode, collision induced dissociation mode and selected reaction monitoring mode of the API 4000 QTrap triple quadrupole instrument (AB Sciex, Darmstadt, Germany).

Parameter	Precursor Ion Scan	Collision Induced Dissociation (CID)	Selected Reaction Monitoring (SRM)
Curtain gas	20 psi	20 psi	20 psi
Collision activated dissociation (CAD)	high	high	high
Ion spray voltage	5500 V	5500 V	5500 V
Temperature	550 °C	550 °C	550 °C
Nebulizer gas	30 psi	30 psi	30 psi
Auxiliary gas	60 psi	60 psi	60 psi
Interface heater	on	on	on
Declustering potential	151 V	151 V	151 V
Entrance potential	10 V	-	10 V
Collision energy	80 V	100 V	100 V
Collision energy spread	-	10 V	-
Exit potential	26 V	-	26 V
